# Investigation into Alternative Sample Preparation Techniques for the Determination of Heavy Metals in Stationary Source Emission Samples Collected on Quartz Filters

**DOI:** 10.3390/s141121676

**Published:** 2014-11-17

**Authors:** Sharon L. Goddard, Richard J. C. Brown

**Affiliations:** Analytical Science Division, National Physical Laboratory, Teddington, Middlesex TW11 0LW, UK; E-Mail: richard.brown@npl.co.uk

**Keywords:** stationary source emissions, heavy metals, quartz filters, hydrofluoric acid, fluoroboric acid, aqua regia

## Abstract

Monitoring stationary source emissions for heavy metals generally requires the use of quartz filters to collect samples because of the high temperature and high moisture sampling environment. The documentary standard method sample preparation technique in Europe, EN 14385, uses digestion in hydrofluoric acid and nitric acid (HF/HNO_3_) followed by complexing with boric acid (H_3_BO_3_) prior to analysis. However, the use of this method presents a number of problems, including significant instrumental drift during analysis caused by the matrix components, often leading to instrument breakdown and downtime for repairs, as well as posing significant health and safety risks. The aim of this work was to develop an alternative sample preparation technique for emissions samples on quartz filters. The alternative techniques considered were: (i) acid digestion in a fluoroboric acid (HBF_4_) and HNO_3_ mixture and (ii) acid extraction in an *aqua regia* (AR) mixture (HCl and HNO_3_). Assessment of the effectiveness of these options included determination of interferences and signal drift, as well as validating the different methods by measurement of matrix certified reference materials (CRMs), and comparing the results obtained from real test samples and sample blanks to determine limits of detection. The results showed that the HBF_4_/HNO_3_ mixture provides the most viable alternative to the documentary standard preparation technique.

## 1. Introduction

Over recent decades, the worldwide requirement for environmental monitoring of industrial pollutant emissions, including for heavy metals, has been increasing prompted by ever tighter legislation governing emissions. Heavy metals are naturally occurring in the environment, but human activities such as mining, metal smelting and refining, and combustion of fossil fuels have significantly increased emissions, and thus population exposure. Studies by the World Health Organisation (WHO) have shown heavy metals to be harmful to human health [1], with long term exposure resulting in neurotoxic and carcinogenic effects. The majority of airborne metals emissions are bound to particulate matter (PM), which has been shown by epidemiological and toxicological studies to have various inflammatory, cytotoxic, mutagenic and carcinogenic effects on the lungs, dependent on the PM size fraction and composition [2]. Airborne PM is also considered a contributing factor in increasing the risk of heart ailments and heart attacks [3].

Industrial pollution control in EU member states is currently governed by the European Industrial Emissions Directive (IED) [4]. The IED covers industrial activities with major pollution potential, including large combustion plants (defined as those whose rated thermal input is equal to or greater than 50 MW), waste incineration/co-incineration plants, metal production and processing facilities, and installations using organic solvents or producing titanium dioxide [4]. The IED sets emission limit values and monitoring requirements for pollutants to air from these processes. In England and Wales the monitoring of stationary source emissions (from sample collection to analysis) must also comply with the quality requirements of the Monitoring Certification Scheme (MCERTS) [5] established by the Environment Agency (EA). In relation to air monitoring, to be eligible to perform sample analysis a testing laboratory must be accredited to ISO 17025 for the relevant standards, as verified by the United Kingdom Accreditation Service (UKAS), and any additional requirements imposed by the MCERTS scheme [6].

To perform heavy metals analysis of stack emissions in Europe, the analytical procedures of the testing laboratory must follow standard method EN 14385, *Stationary source emissions*—*Determination of the total emission of As*, *Cd*, *Cr*, *Co*, *Cu*, *Mn*, *Ni*, *Pb*, *Sb*, *Tl and V* [7] which has been designated as the European reference method. EN 14385 stipulates that quartz filters must be used to sample the stationary source emissions. These filters and the corresponding solutions used to rinse the sampling probes and filter housing shall be initially digested in a matrix of HF, HNO_3_ and then complexed with H_3_BO_3_ prior to analysis. No alternative preparation methods are currently considered appropriate to provide results compliant with these standards, and furthermore no alternatives are allowed by the UK Environment Agency's Method Implementation Document [8] (which applies the additional requirements of the MCERTS scheme).

EN 14385 does not mandate any single analytical method, instead it provides a small number of performance requirements for the analysis; however, it does list atomic absorption spectroscopy (AAS), inductively coupled plasma optical emission spectrometry (ICP-OES), and inductively coupled plasma-mass spectrometry (ICP-MS) as appropriate exemplar techniques. At NPL, analysis of the digested samples is performed by ICP-MS. ICP-OES is significantly less sensitive than ICP-MS for most metals, and AAS, the predecessor to ICP techniques, is limited in the number of metals that can be determined in any one analysis because it requires the use of an individual radiation source, usually a hollow cathode lamp, for each separate analyte [9].

To reduce cost and minimise time on site, sampling tests from stationary sources are often conducted for closer to the minimum allowed 30 min than the maximum 8 h period. As a result very small total masses of metals are collected during sampling. This makes the ICP-MS the preferred technique to analyse these samples because of the high sensitivity previously mentioned. However the post digestion sample matrix as specified in the standard has proved problematic for the ICP-MS to process. Components of this matrix typically precipitate out of solution inside the instrument, leaving deposits on the cones and ion lens that cause significant signal drift and reduced sensitivity. Consequently, the data produced shows relatively high standard deviations, resulting in higher associated analytical uncertainties. Additionally, there is regular instrument downtime for cleaning, and often complete breakdowns requiring expensive engineer support and replacement parts. Some improvements to the methodology have already been implemented [10] to deal with the symptoms of the problem, but these changes were insufficient to deal with the root cause of the problem: the sample matrix.

The purpose of this study is to assess the suitability of alternative sample preparation techniques in order to improve the quality of data produced, reduce the need for extensive instrument maintenance and breakdown support, and risks to health and safety for scientists. Previous attempts have been made to introduce methods that avoid direct handling of HF [11], however this approach required the use of NaF and H_3_BO_3_, both of which would contribute significant extra salt precursors to the matrix. This is not ideal, as this increases the likelihood of matrix-induced interferences and signal drift for ICP-MS analysis. The alternatives investigated here are: (i) acid digestion in a HBF_4_/HNO_3_ mixture and (ii) acid extraction in an AR matrix. Fluoroboric acid has been shown to be effective in digestion of peat and plant matrices for the determination of rare earth elements, an analysis which also needs to dissolve siliceous material [12]. *Aqua regia* is also considered suitable for extracting trace elements from soil samples [13,14], although more conventionally as a preparation for AAS or ICP-OES determinations, so was also investigated. Analytical challenges associated with the matrices including interferences and drift were assessed and, where possible, mitigated. The analytical methods were optimised for the use of these new acid mixtures. Verification of the suitability of these matrices took the form of extraction efficiency tests on certified reference materials (CRMs) recommended in EN 14385 [7], comparing test sample results and monitoring detection limits. It is hoped that if sufficient evidence of a viable alternative is presented, these options may be included in future revisions of the standards.

## 2. Experimental Section

Sub-samples of the recommended certified reference material BCR-038 (fly ash from pulverized coal) [7] (supplied by the European Commission Joint Research Centre Institute for Reference Materials and Measurements) were accurately weighed on a calibrated balance (Sartorius LA230S, resolution 0.1 mg). Filter samples were cut into accurate portions using a template and ceramic scissors. The sub-samples were then transferred to microwave vessels and the acid mixtures added:

(i) 0.2 mL HBF_4_/9.8 mL HNO_3_, or
(ii) 7.5 mL HCl/2.5 mL HNO_3_

The samples were then heated under pressure using a microwave (Anton Paar Multiwave 3000, Graz, Austria). The temperature program, which meets the requirements of EN 14385 [7], heats the samples initially to 180 °C, holds for 15 min at this temperature, then continues to heat to 220 °C and holds for a further 35 min, with power used up to 1400 W. The HCl and HNO_3_ used were concentrated trace analysis grade (Fisher). The HBF_4_ was supplied as a 50% aqueous solution (Apollo Scientific, Stockport, UK). The extracted solutions were diluted in deionised water (18.2 MΩ·cm^−3^, Millipore RiOs/Milli-Q).

An observation during the preparation stages was that the sub-samples of CRM dust material prepared in *aqua regia* did not fully dissolve following the microwave extraction. Therefore the extracted solutions were filtered to remove solids prior to ICP-MS analysis (using Fisherbrand cellulose filter paper). This was necessary to prevent particulates and other undissolved material from clogging in the nebuliser and forming deposits on the cones and lens that would contribute to signal drift. Additionally, it was noted that after digestion of quartz filters with the HBF_4_/HNO_3_ matrix, some filter material remained undigested in the solution. These solutions were also filtered to remove solids prior to ICP-MS analysis as above.

Sample data for the European standard HF/HNO_3_/H_3_BO_3_ matrix were obtained by preparation as detailed in EN 14385 [7]. The samples were digested in a mixture of 2 mL HF (40% analytical reagent grade, Fisher, Loughborough, UK) and 3 mL HNO_3_ (concentrated trace analysis grade, Fisher) in a microwave (CEM MARS, Buckingham, UK). The temperature program meets the requirements of EN 14385 [7], involved a 15 min ramp, heating the samples to 210 °C, followed by a hold time at this temperature for 20 min. The power used was up to 800 W.

After the first digestion the vessels were removed from the microwave and allowed to cool. The vessels were then opened, and 20 mL saturated H_3_BO_3_ (55 g powder dissolved in 1 L deionised water) and 20 mL deionised water were added. The vessels were re-sealed and the digestion program repeated. After this second digestion, the extracted solutions were diluted in deionised water.

The extracted, diluted and filtered sample solutions were analysed for heavy metals as specified in standard EN 14385 [7] using a PerkinElmer Elan DRC II ICP-MS (Seer Green, UK). The ICP-MS was optimised prior to analysis to ensure a combination of sufficient signal and low oxide formation criteria were met. An example of typical optimization parameters are set out in Table 1.

Drift correction was performed by plotting the responses from repeat analyses of a quality assurance (QA) solution containing mid-range concentrations of the analytes under test throughout the analytical sequence. The equation of the fourth order polynomial line fitted though the QA responses was used to correct the responses of the calibration standards and samples, as in previous work [10]. Initial tests also utilised an internal standard solution containing elements other than those specifically under test, and the ratios of each analyte to its relevant internal standard were plotted. However this proved to increase the standard deviation and reduce the accuracy of responses, possibly because these stationary source emissions samples contained significant quantities of the elements used as internal standards. As a result this practice was not used in the analyses detailed here.

The ICP-MS was calibrated by direct analysis of external calibration standards containing known concentrations of the analytes, traceable to the NIST SRM 3100 series of mono-elemental solutions. Calibration curves were generated from the measured responses of the calibration standards and used to interpolate the sample concentrations using NPL's XLGenline software which performs generalised least squares regression [15]. The calibration range encompassed the measured sample concentrations. The calibration standards were prepared in a matrix of 6.9% HNO_3_/3.7% HCl for mercury and 11.2% HNO_3_/1.2% H_2_O_2_ for all other metals. All measurements were blank corrected with the appropriate matrix blank. Sample data for the standard HF/HNO_3_/H_3_BO_3_ matrix were obtained by analysis as detailed in EN 14385 [7]. The ICP-MS was optimised, corrected for drift (also without internal standard) and calibrated as above. The calibration standards were prepared in a matrix of 1.6% HF, 4.2% HNO_3_, 2% H_3_BO_3_ for all metals.

## 3. Results and Discussion

### 3.1. Analytical Challenges

#### 3.1.1. Chloride Interferences

Chloride was present in both proposed matrices: at residual levels in HBF_4_ (from the production process) and as a major component (HCl) in the AR extraction matrix. This introduced chloride-forming polyatomic interferences during ICP-MS determination. Two such interferences are relevant for the analyses described here:

- [ClO]^+^ interfered with vanadium at *m*/*z* 51
- [ArCl]^+^ interfered with arsenic at *m*/*z* 75 (Ar is used to generate the plasma)

The effects of these interferences may be observed as non-linear calibrations, high reagent blank values and high standard deviations from replicate analyses. [ClO]^+^ would also be expected to potentially interfere with determination of Cr, *m*/*z* 52, from the combination of ^35^Cl^17^O. However, the effects as detailed above were less apparent for Cr compared to V and As, probably because the relative natural isotopic abundance (RA) of ^17^O is only 0.038%, so it was decided to focus on corrections for those more significantly affected elements. Mitigation measures included both sourcing reagents with low chloride content and performing mathematical interference corrections.

Sourcing reagents with a lower chloride content, or reducing the volume of the reagent used in sample preparation, minimises the potential for the formation of polyatomic interferences in the ICP-MS plasma.

Stocks of HBF_4_ from three independent suppliers were tested. The 50% HBF_4_ in aqueous solution supplied by Apollo Scientific was found to give the best combination of minimised standard deviations associated with CRM measurements, matrix blank concentrations and detection limits. In addition, a range of HBF_4_: HNO_3_ proportions were tested in order to establish the minimum proportion of HBF_4_ required to produce acceptable CRM recoveries (see Sample preparation, above, for optimised matrix proportions). These optimisations were not performed for the AR matrix, because the chloride content constitutes a much more significant proportion of that particular matrix and as such would show insignificant benefits from such measures.

Mathematical correction of responses based on known RAs of the relevant elements can be an effective tool to remove the influence of some interferences, and resolve potential biases between sample and calibration matrices. It can be simply incorporated as part of the ICP-MS analysis method. The suitability of applying such corrections for V and As was investigated for the AR matrix as this has the highest contribution from chloride interference.

The most abundant naturally occurring V isotope (99.75% RA) has a mass of 51 amu [16]. [ClO]^+^ interferes with the determination of V because the most abundant isotopes of chlorine (^35^Cl) and oxygen (^16^O) combine to give a compound mass of 51 amu. A correction equation was established that compared the ICP-MS signal, factored for differences in RA, from mass 51 to mass 53 (from ^37^Cl^16^O) and accounted for any ^53^Cr by reference to ^52^Cr:

(1) $$I_{51}-\left(3.127\left(I_{53}-\left(0.113I_{52}\right)\right)\right)$$

where *I_x_* would correspond to the analytical intensity measured at mass to charge ratiox. Calibration standards and samples were analysed for V, initially without any correction. After the uncorrected dataset was recorded it was reprocessed with correction Equation (1) and the two sets of data were compared. The dataset processed with the correction showed poorer linearity of the calibration curve in comparison to the uncorrected data. This may be because there was a significant signal from [ClO]^+^ at *m*/*z* 52, despite the low RA of ^17^O, as described above.

Mathematical corrections of this type are of most use when the proportion of the total signal accounted for by interfering ions is relatively low. When the quantity of interfering ions is too high, there may be inconsistent and variable formation rates of the interfering polyatomic compounds. In this eventuality, the contribution to the signal from the interference will be variable, even within the timespan required for replicate readings of the same determination, thereby increasing the uncertainty in the corrected result. Clearly, this would also result in reduced precision between repeat determinations.

As this test showed that employing an interference correction equation did not improve the data quality for V analysis in this reagent matrix, and the proportion of signal attributable to the interferent should be comparable between calibration standards and samples, it was decided to proceed without this measure for subsequent analyses.

The only naturally occurring isotope of As is ^75^As [16]. [ArCl]^+^ interferes with the determination of As because the most abundant isotopes of Ar (mass 40), and Cl (mass 35) combine to give a compound mass of 75. Similarly to the V correction, the As correction is based on the two Cl isotopes. [ArCl]^+^ can be measured at mass 77 because ^40^Ar can combine with both ^35^Cl and ^37^Cl. Again, due to the lower RA of ^37^Cl, the [ArCl]^+^ signal at mass 77 would be a factor of 3.127 lower than the signal at mass 75. So the signal at mass 77 should be multiplied by a factor of 3.127, then this value should be subtracted from the mass 75 signal:

(2) $$I_{75}-\left(3.127I_{77}\right)$$

However selenium has a 7.63% RA isotope at mass 77 which interferes with ^40^Ar^37^Cl. The signal from ^82^Se (8.73% RA) can be used to establish the proportion of signal at mass 77 attributable to ^77^Se:

(3) $$I_{82}\left(\frac{7.63}{8.73}\right)$$

Furthermore, krypton also has an isotope at mass 82 (11.6% RA) [16]. However, tests on the matrix blank showed no significant difference between omission or inclusion of the additional correction factor for Kr, suggesting the signal was negligible in this matrix. So the final correction equation for As is:

(4) $$I_{75}-\left(3.127\left(I_{77}-\left(0.874I_{82}\right)\right)\right)$$

**Figure 1.**
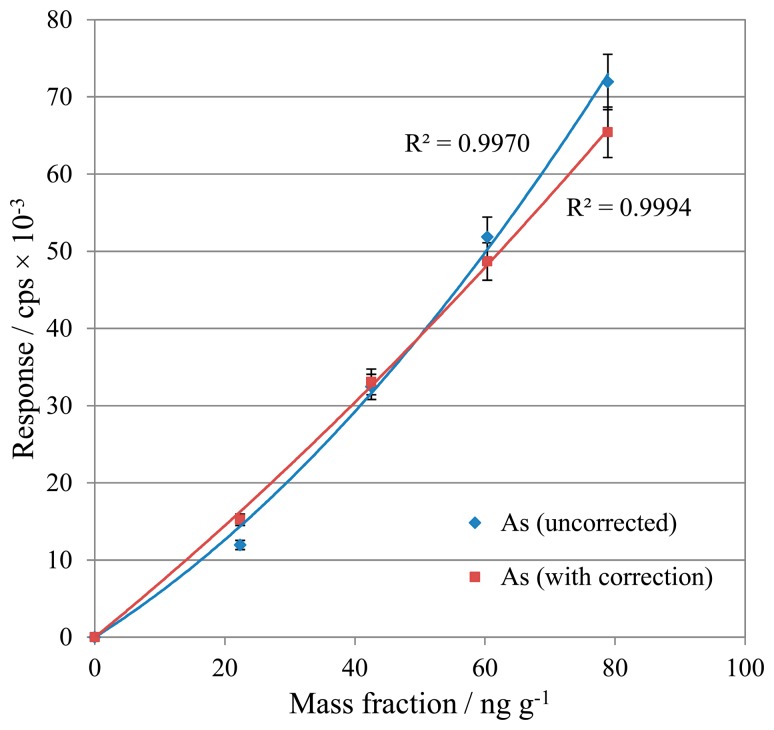
Arsenic calibrations with and without interference correction, response as counts per second (cps) × 10^−3^. Calibration curves are fitted second order polynomials. The errors bars represent the standard deviation of five repeat measurements of each standard.

Calibration standards and samples were analysed for As, initially without any correction, then with correction Equation (4). The two sets of data are compared in Figure 1.

In contrast to V, the As calibration demonstrated an improvement in linearity with the application of the interference correction. This suggests that the relatively low reactivity of Ar resulted in minimal [ArCl]^+^ formation, for which the interference correction was able to compensate successfully. From these tests, interference correction is demonstrated to provide a beneficial method addition for the processing of As results, so this correction equation was retained for future work.

#### 3.1.2. Signal Drift

Drift in analytical sensitivity not only suggests that the measurement is not under full control; additionally, EN 14385 specifies a maximum of 10% drift within an analytical run [7], otherwise the problem causing the drift needs correcting before the samples can be re-analysed. The sample matrix can introduce or exacerbate the potential for signal drift. This can impose limits on the maximum length of analytical runs, making the overall analytical process lengthier and more costly. For these reasons the use of acid mixtures which limit analytical drift is highly desirable.

In an analysis where signal drift is understood and under control, sample responses can still be corrected for drift by regular measurements of a QA standard solution [10]. Significant signal drift is a known problem for the standard HF/HNO_3_ mixture and potentially a problem for the two new digestion mixtures proposed, due to the various combinations of salt precursors and relatively high ionic content.

**Figure 2.**
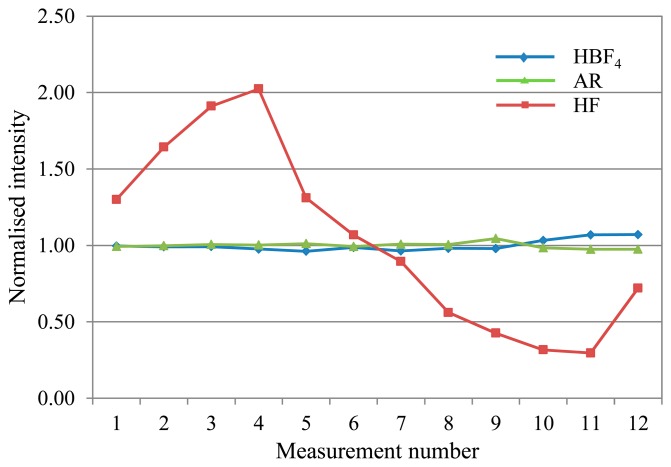
Vanadium drift plot, showing typical deviation trends of the three matrices representative of majority of analytes considered.

**Figure 3.**
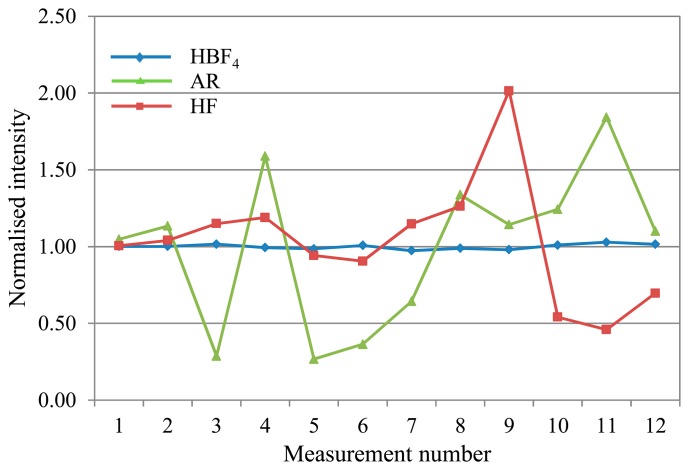
Arsenic drift plot, showing severe drift in both the AR matrix (from the [ArCl]^+^ interference) and the standard HF matrix.

**Figure 4.**
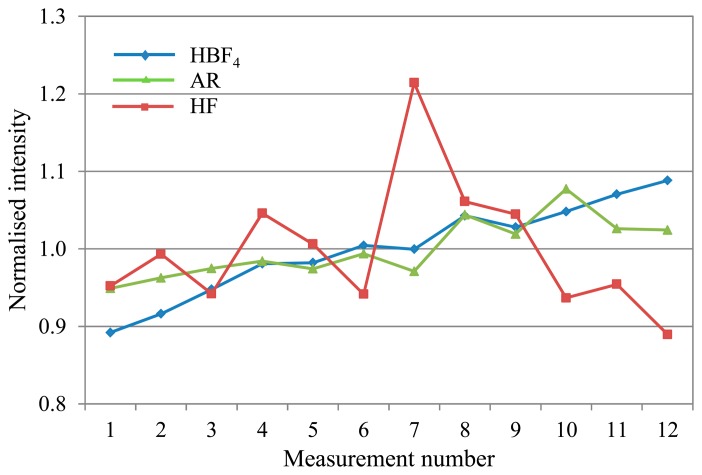
Mercury drift plot, showing anomalous drift in the HF matrix.

(1) Standard matrix: HF/HNO_3_/H_3_BO_3_
  The standard preparation of HF/HNO_3_ requires addition of H_3_BO_3_ to complex with insoluble complex fluorides forming soluble fluoroborates [17], making the resulting solution safer to handle and reducing the risk of damage to analytical instrumentation. With a stoichiometric amount ratio of at least 4:1 HF: H_3_BO_3_, the result is formation of HBF_4_ over a two-stage exothermic reaction [18]:
  (5) $$H_{3}BO_{3}+3HF\to HBF_{3}+2H_{2}O$$
  (6) $$HBF_{3}OH+HF\to HBF_{4}+H_{2}O$$
  In the initial digestion, 2 mL concentrated HF and 3 mL concentrated HNO_3_ equates to 9.28 M HF. Subsequent addition of 20 mL 5% H_3_BO_3_ and 20 mL deionised water gives 1.03 M HF + 0.50 M H_3_BO_3_, so the amount ratio is 2:1 HF: H_3_BO_3_. Therefore 0.26 M HBF_4_ is produced in the final sample matrix. As the H_3_BO_3_ is added in stoichiometric excess to ensure all the fluoride ions are complexed, the resulting sample matrix has a high total dissolved solids content that tends to precipitate out of solution inside the ICP-MS [19], causing drift.
  This sample matrix also requires additional heating (following addition of the H_3_BO_3_ and water) above 130 °C (boiling point of HBF_4_) to decompose the transition metal fluoroborates formed with the metal analytes [20]. Such salt complexes would also be liable to precipitate out of solution [19], contributing both to drift and interferences, as well as reduction in the analyte signal if the analyte ions have formed salt compounds and thus are not detected at their elemental ionic mass. The acid blank solution used to prepare the calibration standards also requires heating. Non-linear calibration curves are obtained if the acid solution is not heated before it is used for standard preparation.
(2) Alternative matrix: HBF_4_/HNO_3_
  In the alternative matrix of HBF_4_/HNO_3_, 0.2 mL concentrated HBF_4_ is used in the digest. Once diluted, the result is only 0.03 M HBF_4_ in the sample matrix, so a much reduced salt-producing capacity in comparison to the standard preparation. Production of the 50% in aqueous solution HBF_4_ stock does incorporate up to a few percent excess H_3_BO_3_ to prevent risk of HF formation over time as HBF_4_ hydrolyses [20], so this will contribute to some additional salt formation. Linear calibrations have been obtained without heating the acid matrix prior to calibration standard preparation, supporting the supposition that the lower content of salt-forming compounds results in fewer interferences. However, the accuracy has not yet been cross-verified with CRMs measured with matrix-matched calibration standards. Independent measurement of CRMs has yielded good recoveries, and linear calibrations have been obtained separately with matrix-matched standards.
(3) Alternative matrix: AR
  The AR matrix seems to have the least potential for salt precipitation of the three matrices considered. However the chloride does form significant polyatomic interferences with the Ar carrier gas and with oxygen in the aqueous sample solution as discussed above. The [ClO]^+^ interference does not seem to adversely impact the V drift, but the [ArCl]^+^ is a problem for As drift correction. Despite the use of corrections for these interferences the standard deviation of the responses remains high in absolute terms which in turn has an adverse effect on the limits of detection for As and V when using the AR mixture.
(4) Matrix drift comparison
  In comparing drift for the three mixtures during a 4.5 h analysis, the majority of analytes showed manageable drift in the range of approximately 0.85–1.15 (normalised to the average intensity for the run) for the proposed alternative matrices. In contrast, the standard HF matrix exhibited significant drift for most analytes, with intensity ratios typically ranging from a peak over 2 early on in the run, before falling away to less than 0.5 by the end. Figure 2 shows typical deviation trends for each of the three matrices as observed with V.
  Arsenic (see Figure 3), displayed significant variability in the range 0.20–1.85 with the AR matrix, as explained above. The HF matrix showed a similar deviation range to the AR, in a change from its characteristic drift pattern as seen in Figure 2.
  Mercury (see Figure 4), also showed short-term variability with the standard HF matrix.
  Overall, the HBF_4_/HNO_3_ matrix shows the least and most predictable, and therefore correctable, deviation across an analytical run for most analytes.

### 3.2. CRM Recoveries

**Figure 5.**
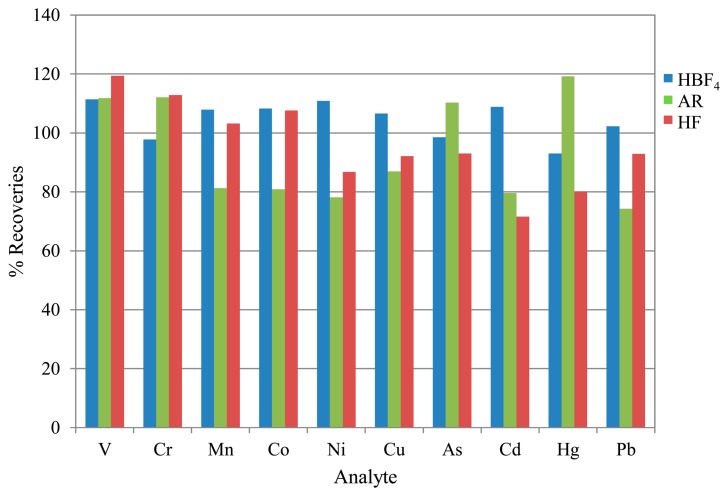
Comparison of the average recoveries obtained from the CRM BCR-038 using HBF_4_ digestion, AR extraction and HF digestion (standard method). Indicative values only were given for V and Ni.

Portions of the CRM recommended in standard EN 14385, BCR-038, were prepared and analysed as specified in the Experimental section. A summary of the results is shown in Figure 5.

The resulting recoveries were within acceptable parameters and comparable to typical recoveries obtained using the standard method of HF digestion or better, showing that both alternative preparation techniques can be considered efficient extraction matrices. However, it can be seen that recoveries are lower for the majority of analytes using the AR extraction. In addition, the high uncertainties associated with V and As suggest a significant interference effect from the HCl (see Table 2 above).

Overall, the HBF_4_ digestion gives the most accurate average results, with both HF and AR techniques showing reduced recoveries from the mid to high mass range. Additionally, it should be noted that uncertainties associated with the V and As recoveries for the AR matrix were in the region of 100% (*k* = 2), based on standard deviations of replicate determinations in combination with calibration standard preparation uncertainties. This is attributable to the chloride interferences.

### 3.3. Test Samples

**Figure 6.**
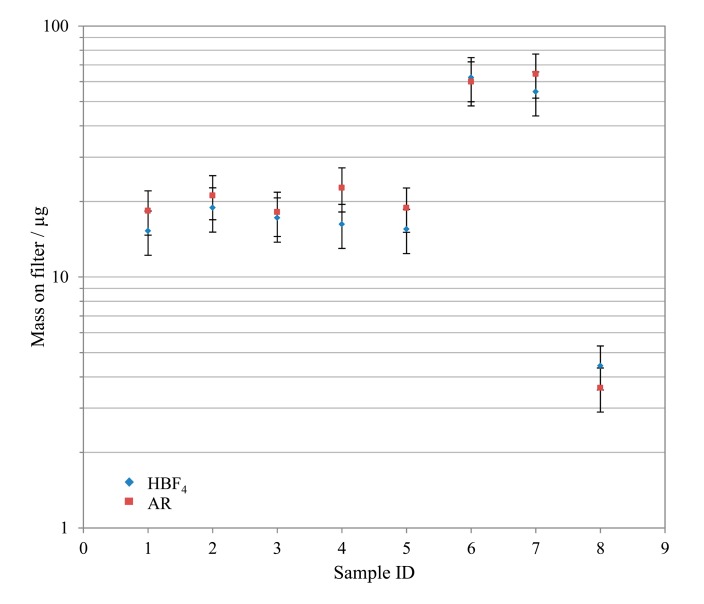
Comparison of the mass of Pb observed on quartz filters using the HBF_4_/HNO_3_ and AR extraction techniques, error bars set to 20%.

A number of real quartz filter samples taken from a waste incinerator in south east England were prepared according to both alternative preparation techniques. In general, the observed mass on filter results for both preparations agreed to within ±20%, with the exception of levels approaching the detection limit. Figure 6 shows an example of this good comparability for Pb.

### 3.4. Limits of Detection

The method detection limit for each analyte is based on multiple analyses of the blank matrix solution calculated as specified in EN 14902 [21].

As can be seen in Table 3, the limits of detection for V and As are significantly higher with the AR matrix due to the chloride interference. The HBF_4_/HNO_3_ matrix has higher V, Cr, Mn, Hg and Pb detection limits than both the AR and HF matrices. With these exceptions, detection limits for both the alternative matrices are either comparable to or better than those achieved with the standard method (HF). Both show significant improvements for Ni, Cd and Tl over the standard method.

For reporting purposes, the limits of detection (LODs) are based on multiple analyses of blank filters (quarter or half portions of specified diameter quartz filters). However the filter LODs listed in Tables 4 and 5 below should only be considered as indicative, as they are averaged from sub-samples of varying size. Stack emissions can be sampled onto filters of various diameters, from which different portions are sub-sampled thereof (considering what area of filter can reasonably be expected to be extracted by 10 mL acid). The results were normalised for the total and sub-sampled filter areas, but ideally, further tests should be performed to verify separate LODs for all the different permutations of filter and sub-sample portion sizes that could be used.

The filter sub-sample is digested in the appropriate acid matrix, then corrected to account for the sub-sampling and any dilutions to which the samples were subjected in the preparation stage. To be fit for purpose, the dilution-corrected limits of detection, when converted to a concentration in air, need to be below the IED limit values (0.05 mg·m^−3^ (standardised for temperature and pressure) for Cd and Tl combined, 0.05 mg·m^−3^ for Hg, and 0.5 mg·m^−3^ for the sum of Sb, As, Pb, Cr, Co, Cu, Mn, Ni, V) [4]. To ensure that any result at or above the IED limit values would be detected, the dilution-corrected method limits of detection were converted to their maximum theoretical concentration in emissions, based on a minimum sampling time of 30 min at a minimum flow rate of 1 m^3^·hr^−1^:

(7) $$\text{LOD}\left(\text{emissions}\right)=\frac{{\text{LOD}}_{m}.m}{v}$$

where:
*LOD_m_* is the method detection limit (dilution corrected)/μg·g^−1^*m* is the mass of diluted sample/g*v* is the minimum volume of air sampled/m^3^

In EN 14385 [7], the maximum permissible detection limit for each element from the entire sampling train is 5 μg·m^−3^. As the HF digestion preparation has been shown to meet this criterion, and the LODs obtained with the alternative methods will also meet this limit given that they show very similar values.

Table 5 shows that the theoretical maximum limit of detection values for all analytes, summed as specified in the IED Directive, were significantly less than their corresponding limit values—at least two orders of magnitude in most cases. Therefore it may be confidently accepted that any sample of a concentration at or above the IED limit value, prepared with either of the proposed techniques, will be detected and reported.

## 4. Conclusions

This report has proposed two alternative sample preparation techniques (HBF_4_/HNO_3_ digestion and extraction with AR) to the technique described in EN 14385 [7] (digestion with HF) for the preparation of stationary source emission samples.

All the matrices being considered have particular analytical challenges associated with them, including the EN 14385 standard HF method. The advantages and disadvantages of each were identified and assessed in terms of their impact on the quality of analytical results. A summary of the comparison of the three acid mixtures for sample preparation is shown in Table 6.

The AR preparation introduces spectral interferences that arise from the chloride in the matrix and significantly affect the measurement of V and As. Mitigation measures including application of an interference correction calculation on the As measurement and reducing the nebuliser flow rate have been shown to reduce the impact of these interferences. However this interference has a significantly detrimental effect on the signal stability of As, such that the drift correction as described would provide insufficient compensation.

Salt formation was a problem experienced with both the standard method (HF/HNO_3_/H_3_BO_3_) and the HBF_4_/HNO_3_ matrix. The fluoride and H_3_BO_3_ components had a tendency to bond with the metal analytes to form salts [20], which would precipitate out of solution when subjected to the high temperatures in the Ar plasma of the ICP-MS [19]. These salts would then coat the ICP-MS cones, resulting in increased signal drift and the potential for interferences. An additional consequence would be a reduction in the analyte signal, assuming some of the analyte ions had formed salt compounds, so would not be detected at their mass to charge ratio. Since the HBF_4_/HNO_3_ matrix contained much lower levels of salt precursors, it was found to be the least affected in terms of signal drift. Even with this matrix it is worth keeping run times to a minimum, and cleaning the cones after every run in order to curtail salt accumulation. Linear calibrations have been achieved with standards prepared in the HBF_4_/HNO_3_ matrix, suggesting that heating the matrix solution is not necessary in order to hydrolyse the fluoroboric ions to prevent interference formation, as it is with the standard preparation.

It has been established that both alternative preparation methods can achieve quantitative recoveries of the CRM recommended in EN 14385 [7], comparable or better to those obtained by the standard method of HF digestion. Recoveries with the HBF_4_/HNO_3_ matrix were the most consistently accurate across the mass range.

Testing of real filter samples showed good agreement between the HBF_4_/HNO_3_ digestion and AR extraction methods. Due to insufficient sample availability, test filter sample results were only compared between the two alternative methods, so future work should ideally include a comparison of sample results obtained with the standard method as well. Also, no filter rinsing solutions were tested with the new methods, so further tests should be conducted to ensure comparability of results can be achieved for this sample type.

Limits of detection have been determined and assessed as likely to be fit-for-purpose by comparison with the IED limit values [4] for both alternative matrices, subject to a thorough investigation of concentrations in the various sub-sampling portions.

Overall, this work has shown that the proposed preparation technique of HBF_4_/HNO_3_ digestion currently offers the most viable alternative to the standard method of digestion with HF, while offering a much reduced health and safety risk to scientists. We propose that this alternative sample preparation method should be considered for inclusion when the current EN14385 standard is revised.

## Figures and Tables

**Table 1.** PerkinElmer Elan DRC II typical optimisation parameters.

**Optimisation Parameter**	**Setting**
Nebuliser Flow (L/min)	0.87–0.93
Lens Voltage (V)	Mg 4.25–5.75, U 7.25–9.25
Power (W)	1250
Auxiliary Gas Flow (L/min)	1.20
Plasma Gas Flow (L/min)	15.00
Analog Stage Voltage (V)	−1950
Pulse Stage Voltage (V)	1100
Quadrupole Rod Offset Std [QRO] (V)	0.00
Cell Rod Offset Std [CRO] (V)	−12.00
Cell Path Voltage Std [CPV] (V)	−11.00
Discriminator Threshold (V)	25.00

**Table 2.** Comparison of % recoveries obtained with all three preparation methods from BCR-038. EN 14385 [7] stipulates that measured concentrations shall not differ more than 10% from the certified value or shall be within twice the uncertainty value quoted for the CRM, whichever is larger (* V and Ni acceptable ranges are shown as ‘N/A’ because BCR-038 gives indicative values only for these elements). In the UK, MID 14385 [8] allows for recoveries 100% ± 20%.

**Analyte**	**Mean Recovery / %**			**Acceptable Range / %**

	**HBF_4_ Matrix**	**AR Matrix**	**HF Matrix (EN 14385)**	
V	111	112	119	N/A *
Cr	98	112	113	90–110
Mn	108	81	103	90–110
Co	108	81	108	90–110
Ni	111	78	87	N/A *
Cu	107	87	92	90–110
As	99	110	93	90–110
Cd	109	80	72	87–113
Hg	93	119	80	86–114
Pb	102	74	93	90–110

**Table 3.** Comparison of LODs obtained with both preparation methods.

**Analyte**	**LOD/ng·g^−1^**		

	**HBF_4_ Matrix**	**AR Matrix**	**HF Matrix (EN 14385)**
V	2.08	60.7	1.26
Cr	9.79	5.60	0.349
Mn	7.85	0.152	4.57
Co	0.373	0.002	0.357
Ni	0.353	0.141	1.74
Cu	1.10	0.045	2.92
As	0.430	2.28	0.296
Cd	0.018	0.001	0.037
Sb	0.243	0.018	0.136
Hg	0.204	0.018	0.060
Tl	0.001	0.004	0.014
Pb	5.39	0.013	0.432

**Table 4.** Maximum LOD values (filters) expressed as concentrations of analyte in air.

**Analyte**	**Concentration of Analyte in Emissions / μg·m^−3^**		

	**HBF_4_ Digestion**	**AR Extraction**	**HF Digestion (EN 14385)**
V	0.555	3.453	0.662
Cr	3.764	1.955	1.661
Mn	2.838	0.475	0.409
Co	0.170	0.055	0.032
Ni	1.260	0.618	2.140
Cu	3.008	1.982	3.044
As	0.315	0.539	0.759
Cd	0.128	0.004	0.017
Sb	1.457	0.030	0.052
Hg	0.073	0.008	0.056
Tl	0.020	0.004	0.004
Pb	1.057	0.222	1.033

**Table 5.** Comparison of emission detection limits with IED limit values [4].

	**Concentration of Analyte in Emissions/μg**·**m^−3^**			

	**HBF_4_ Digestion**	**AR Extraction**	**HF Digestion (EN 14385)**	**IED Limit Values [4]**
Sum V-Pb	14.42	9.33	9.792	500
Sum Cd, Tl	0.148	0.008	0.021	50
Hg	0.073	0.008	0.056	50

**Table 6.** Summary comparison of the attributes of the three acid mixtures for sample preparation tested in this paper ranked with performance decreasing in the order: 1–3.

**Acid Mixture**	**Drift**	**CRM Recovery**	**LOD**
**HBF_4_ digestion**	1	1	2
**AR extraction**	2	3	3
**HF digestion (EN 14385)**	3	2	1
